# Classes of Int-Soft Filters in Residuated Lattices

**DOI:** 10.1155/2014/595160

**Published:** 2014-08-27

**Authors:** Young Bae Jun, Sun Shin Ahn, Kyoung Ja Lee

**Affiliations:** ^1^Department of Mathematics Education, Gyeongsang National University, Jinju 660-701, Republic of Korea; ^2^Department of Mathematics Education, Dongguk University, Seoul 100-715, Republic of Korea; ^3^Department of Mathematics Education, Hannam University, Daejeon 306-791, Republic of Korea

## Abstract

The notions of int-soft filters, int-soft *G*-filters, regular int-soft filters, and *MV*-int-soft filters in residuated lattices are introduced, and their relations, properties, and characterizations are investigated. Conditions for an int-soft filter to be an int-soft *G*-filter, a regular int-soft filter, or an *MV*-int-soft filter are provided. The extension property for an int-soft *G*-filter is discussed. Finally, it is shown that the notion of an *MV*-int-soft filter coincides with the notion of a regular int-soft filter in *BL*-algebras.

## 1. Introduction

In order to deal with fuzzy and uncertain information, nonclassical logic has become a formal and useful tool. As the semantic systems of nonclassical logic systems, various logical algebras have been proposed. Residuated lattices are important algebraic structures which are basic of *MTL*-algebras, *BL*-algebras, *MV*-algebras, Gödel algebras, *R*_0_-algebras, lattice implication algebras, and so forth. The (fuzzy) filter theory in the logical algebras has an important role in studying these algebras and completeness of the corresponding nonclassical logics, and it is studied in [1–8]. Uncertainty is an attribute of information. As a new mathematical tool for dealing with uncertainties, Molodtsov [9] introduced the concept of soft sets. Since then several authors studied (fuzzy) algebraic structures based on soft set theory in several algebraic structures. Acar et al. [10] introduced initial concepts of soft rings. Ahn et al. [11] introduced the notion of int-soft filters of a *BE*-algebra and investigated related properties. They also discussed characterization of an int-soft filter and solved the problem of classifying int-soft filters by their *γ*-inclusive filter. Aktas and Çagman [12] defined soft groups and derived their basic properties using Molodtsov's definition of the soft sets. Atagün and Sezgin [13] introduced and studied soft subrings and soft ideals of a ring by using Molodtsov's definition of the soft sets. Moreover, they introduced soft subfields of a field and soft submodule of a left R-module and investigated some related properties about soft substructures of rings, fields, and modules. Çağman and Enginoğlu [14] constructed a uni-int decision making method which selects a set of optimum elements from the alternatives. Feng et al. [15] improved and further extended Çağman and Enginoğlu's uni-int decision making method in virtue of choice value soft sets and k-satisfaction relations. Çağman and Enginoğlu [16] discussed fuzzy parameterized (FP) soft sets and their related properties and proposed a decision making method based on FP-soft set theory. Feng [17] considered the application of soft rough approximations in multicriteria group decision making problems. Feng et al. [18] initiated the study of soft semirings by using the soft set theory. Jun et al. applied the notion of soft sets by Molodtsov to the theory of *BCK*/*BCI*-algebras, *d*-algebras, and subtraction algebras (see [19–22]). Jun et al. [23] discussed (strong) intersection-soft filters in *R*_0_-algebras. Zhan and jun [24] investigated characterizations of (implicative, positive implicative, and fantastic) filteristic soft *BL*-algebras by means of ∈-soft sets and *q*-soft sets. Recently, Feng and Li [25] explored some relationships among five different types of soft subsets and investigated free soft algebras with respect to soft product operations. They pointed out that soft sets have some nonclassical algebraic properties which are distinct from those of crisp sets or fuzzy sets.

In this paper, we introduce the notions of int-soft filters, int-soft *G*-filters, regular int-soft filters, and *MV*-int-soft filters in residuated lattices and investigate their relations and properties. We consider characterizations of int-soft filters, int-soft *G*-filters, regular int-soft filters, and *MV*-int-soft filters. We provide conditions for an int-soft filter to be an int-soft *G*-filter, a regular int-soft filter, or an *MV*-int-soft filter. We establish the extension property for an int-soft *G*-filter. Finally, we show that the notion of an *MV*-int-soft filter coincides with the notion of a regular int-soft filter in *BL*-algebras.

## 2. Preliminaries

**Definition 1 (see [1, 26, 27]).**

A residuated lattice is an algebra (*L*, ∨, ∧, ⊙, →, 0,1) of type (2,2, 2,2, 0,0) such that

(1) (*L*, ∨, ∧, 0,1) is a bounded lattice;
(2) (*L*, ⊙, 1) is a commutative monoid;
(3) ⊙ and → form an adjoint pair; that is,
  (1) $$\begin{array}{c} \left(\forall x,y,z\in L\right)\;\left(x\leq y⟶z⟺x⊙y\leq z\right). \end{array}$$

In a residuated lattice *L*, the ordering ≤ and negation ¬ are defined as follows:

(2) (∀x,y∈L) (x≤y⟺x∧y=x⟺x∨y=y⟺x⟶y=1)

and ¬*x* = *x* → 0 for all *x* ∈ *L*.

**Proposition 2 (see [1, 2, 6, 7, 26, 27]).**

In a residuated lattice *L*, the following properties are valid:

(3) 1⟶x=x,  x⟶1=1,  x⟶x=1,0⟶x=1,  x⟶(y⟶x)=1.

(4) $$\begin{array}{c} y\leq \left(y⟶x\right)⟶x, \end{array}$$

(5) $$\begin{array}{c} x\leq y⟶z⟺y\leq x⟶z, \end{array}$$

(6) $$\begin{array}{c} x⟶\left(y⟶z\right)=\left(x⊙y\right)⟶z=y⟶\left(x⟶z\right), \end{array}$$

(7) $$\begin{array}{c} x\leq y⟹z⟶x\leq z⟶y,\;\;y⟶z\leq x⟶z, \end{array}$$

(8) $$\begin{array}{c} z⟶y\leq \left(x⟶z\right)⟶\left(x⟶y\right), \end{array}$$

(9) $$\begin{array}{c} \left(x⟶y\right)⊙\left(y⟶z\right)\leq x⟶z, \end{array}$$

(10) $$\begin{array}{c} \neg x=\neg \neg \neg x,\;\;x\leq \neg \neg x, \end{array}$$

(11) $$\begin{array}{c} \neg x\wedge \neg y=\neg \left(x\vee y\right), \end{array}$$

(12) $$\begin{array}{c} x\vee \neg x=1⟹x\wedge \neg x=0, \end{array}$$

(13) $$\begin{array}{c} x⊙y\leq x\wedge y, \end{array}$$

(14) $$\begin{array}{c} x\leq y⟹x⊙z\leq y⊙z, \end{array}$$

(15) $$\begin{array}{c} y⟶z\leq x\vee y⟶x\vee z, \end{array}$$

(16) $$\begin{array}{c} x⟶\left(y\wedge z\right)=\left(x⟶y\right)\wedge \left(x⟶z\right), \end{array}$$

(17) $$\begin{array}{c} x\leq y⟶\left(x⊙y\right). \end{array}$$

**Definition 3 (see [5]).**

A nonempty subset *F* of a residuated lattice *L* is called a filter of *L* if it satisfies the conditions

(18) $$\begin{array}{c} \left(\forall x,y\in L\right)\;\left(x,y\in F⟹x⊙y\in F\right),\\ \left(\forall x,y\in L\right)\;\left(x\in F,x\leq y⟹\;y\in F\right). \end{array}$$

**Proposition 4 (see [5]).**

A nonempty subset *F* of a residuated lattice *L* is a filter of *L* if and only if it satisfies

(19) $$\begin{array}{c} 1\in F, \end{array}$$

(20) $$\begin{array}{c} \left(\forall x\in F\right)\left(\forall y\in L\right)\;\left(x⟶y\in F⟹y\in F\right). \end{array}$$

A soft set theory is introduced by Molodtsov [9], and Çağman and Enginoğlu [14] provided new definitions and various results on soft set theory.

In what follows, let *U* be an initial universe set and let *E* be a set of parameters. Let *P*(*U*) denote the power set of *U* and *A*, *B*, *C*, ⋯⊆*E*.

**Definition 5 (see [9, 14]).**

A soft set $\left(\tilde{f},A\right)$ over *U* is defined to be the set of ordered pairs

(21) $$\begin{array}{c} \left(\tilde{f},A\right)∶=\left\{\left(x,\tilde{f}\left(x\right)\right):x\in E,\tilde{f}\left(x\right)\in P\left(U\right)\right\}, \end{array}$$

where $\tilde{f}:E\to P(U)$ such that $\tilde{f}(x)=\emptyset$ if *x* ∉ *A*.

## 3. Int-Soft Filters

In what follows, we take a residuated lattice *L* as a set of parameters.

**Definition 6 .**

A soft set $\left(\tilde{f},L\right)$ over *U* is called an int-soft filter of *L* if it satisfies

(22) $$\begin{array}{c} \left(\forall x,y\in L\right)\;\left(x\leq y⟹\tilde{f}\left(x\right)\subseteq \tilde{f}\left(y\right)\right), \end{array}$$

(23) $$\begin{array}{c} \;\left(\forall x,y\in L\right)\;\left(\tilde{f}\left(x\right)\cap \tilde{f}\left(y\right)\subseteq \tilde{f}\left(x⊙y\right)\right). \end{array}$$

**Proposition 7 .**

Every int-soft filter $\left(\tilde{f},L\right)$ of *L* satisfies

(24) $$\begin{array}{c} \left(\forall x\in L\right)\;\left(\tilde{f}\left(x\right)\subseteq \tilde{f}\left(1\right)\right), \end{array}$$

(25) $$\begin{array}{c} \left(\forall x,y\in L\right)\;\left(\tilde{f}\left(x\right)\cap \tilde{f}\left(x⟶y\right)\subseteq \tilde{f}\left(y\right)\right). \end{array}$$

**Proof**

Let *x*, *y* ∈ *L*. Since *x* ≤ 1, we have $\tilde{f}(x)\subseteq \tilde{f}(1)$ by (22). Since *x*⊙(*x* → *y*) ≤ *y*, it follows from (23) and (22) that

(26) $$\begin{array}{c} \tilde{f}\left(x\right)\cap \tilde{f}\left(x⟶y\right)\subseteq \tilde{f}\left(x⊙\left(x⟶y\right)\right)\subseteq \tilde{f}\left(y\right). \end{array}$$

This completes the proof.

**Lemma 8 .**

Let $\left(\tilde{f},L\right)$ be a soft set over *U* that satisfies two conditions (24) and (25). Then one has

(27) $$\begin{array}{c} \left(\forall x,y,z\in L\right)\;\left(x\leq y⟶z⟹\tilde{f}\left(x\right)\cap \tilde{f}\left(y\right)\subseteq \tilde{f}\left(z\right)\right), \end{array}$$

(28) $$\begin{array}{c} \left(\forall x,y,z\in L\right)\;\left(x⊙y\leq z⟹\tilde{f}\left(x\right)\cap \tilde{f}\left(y\right)\subseteq \tilde{f}\left(z\right)\right). \end{array}$$

**Proof**

Let *x*, *y*, *z* ∈ *L* be such that *x* ≤ *y* → *z*. Then *x* → (*y* → *z*) = 1, and so

(29) f~(x)∩f~(y)=(f~(x)∩f~(1))∩f~(y)=(f~(x)∩f~(x⟶(y⟶z)))∩f~(y)⊆f~(y)∩f~(y⟶z)⊆f~(z).

Since *x* ≤ *y* → *z*⇔*x*⊙*y* ≤ *z*, (28) is from (27).

**Theorem 9 .**

A soft set $\left(\tilde{f},L\right)$ over *U* is an int-soft filter of *L* if and only if it satisfies two conditions (24) and (25).

**Proof**

The necessity is from Proposition 7.

Conversely, let $\left(\tilde{f},L\right)$ be a soft set over *U* that satisfies (24) and (25). If *x* ≤ *y*, then *x* → *y* = 1 and so

(30) $$\begin{array}{c} \tilde{f}\left(x\right)=\tilde{f}\left(x\right)\cap \tilde{f}\left(1\right)=\tilde{f}\left(x\right)\cap \tilde{f}\left(x⟶y\right)\subseteq \tilde{f}\left(y\right). \end{array}$$

Since *x*⊙*y* ≤ *x*⊙*y* for all *x*, *y* ∈ *L*, it follows from (28) that $\tilde{f}(x)\cap \tilde{f}(y)\subseteq \tilde{f}(x⊙y)$ for all *x*, *y* ∈ *L*. Therefore $\left(\tilde{f},L\right)$ is an int-soft filter of *L*.

**Theorem 10 .**

A soft set $\left(\tilde{f},L\right)$ over *U* is an int-soft filter of *L* if and only if it satisfies condition (27).

**Proof**

The necessity is from Lemma 8 and Theorem 9.

Conversely, let $\left(\tilde{f},L\right)$ be a soft set over *U* satisfying (27). Since *x* ≤ *x* → 1 and *x* → *y* ≤ *x* → *y* for all *x*, *y* ∈ *L*, it follows from (27) that $\tilde{f}(x)=\tilde{f}(x)\cap \tilde{f}(x)\subseteq \tilde{f}(1)$ and $\tilde{f}(x)\cap \tilde{f}(x\to y)\subseteq \tilde{f}(y)$ for all *x*, *y* ∈ *L*. Hence $\left(\tilde{f},L\right)$ is an int-soft filter of *L* by Theorem 9.

**Theorem 11 .**

A soft set $\left(\tilde{f},L\right)$ over *U* is an int-soft filter of *L* if and only if $\left(\tilde{f},L\right)$ satisfies condition (24) and

(31) (∀x,y,z∈L) (f~(x⟶(y⟶z))∩f~(y) ⊆f~(x⟶z)).

**Proof**

Assume that $\left(\tilde{f},L\right)$ is an int-soft filter of *L*. Then condition (24) is valid. Using (6) and (25), we have

(32) f~(x⟶z)⊇f~(y)∩f~(y⟶(x⟶z))=f~(y)∩f~(x⟶(y⟶z))

for all *x*, *y*, *z* ∈ *L*.

Conversely, let $\left(\tilde{f},L\right)$ be a soft set over *U* satisfying (24) and (31). Taking *x*∶ = 1 in (31) and using (3), we get

(33) f~(z)=f~(1⟶z)⊇f~(1⟶(y⟶z))∩f~(y)=f~(y⟶z)∩f~(y)

for all *y*, *z* ∈ *L*. Thus $\left(\tilde{f},L\right)$ is an int-soft filter of *L* by Theorem 9.

**Lemma 12 .**

Every int-soft filter $\left(\tilde{f},L\right)$ over *U* satisfies the following condition:

(34) $$\begin{array}{c} \left(\forall a,x\in L\right)\;\left(\tilde{f}\left(a\right)\subseteq \tilde{f}\left(\left(a⟶x\right)⟶x\right)\right). \end{array}$$

**Proof**

If we take *y*∶ = (*a* → *x*) → *x* and *x*∶ = *a* in (25), then

(35) f~((a⟶x)⟶x)⊇f~(a)∩f~(a⟶((a⟶x)⟶x))=f~(a)∩f~((a⟶x)⟶(a⟶x))=f~(a)∩f~(1)=f~(a).

This completes the proof.

**Theorem 13 .**

A soft set $\left(\tilde{f},L\right)$ over *U* is an int-soft filter of *L* if and only if it satisfies the following conditions:

(36) $$\begin{array}{c} \left(\forall x,y\in L\right)\;\left(\tilde{f}\left(x\right)\subseteq \tilde{f}\left(y⟶x\right)\right), \end{array}$$

(37) (∀x,a,b∈L) (f~(a)∩f~(b) ⊆f~((a⟶(b⟶x))⟶x)).

**Proof**

Assume that $\left(\tilde{f},L\right)$ is an int-soft filter of *L*. Using (3), (24), and (25), we have

(38) f~(y⟶x)⊇f~(x)∩f~(x⟶(y⟶x))=f~(x)∩f~(1)=f~(x)

for all *x*, *y* ∈ *L*. Using (31) and (34), we get

(39) f~((a⟶(b⟶x))⟶x) ⊇f~((a⟶(b⟶x))⟶(b⟶x))∩f~(b) ⊇f~(a)∩f~(b)

for all *a*, *b*, *x* ∈ *L*.

Conversely, let $\left(\tilde{f},L\right)$ be a soft set over *U* satisfying two conditions (36) and (37). If we take *y*∶ = *x* in (36), then $\tilde{f}(x)\subseteq \tilde{f}(x\to x)=\tilde{f}(1)$ for all *x* ∈ *L*. Using (37) induces

(40) f~(y)=f~(1⟶y)=f~(((x⟶y)⟶(x⟶y))⟶y)⊇f~(x⟶y)∩f~(x)

for all *x*, *y* ∈ *L*. Therefore $\left(\tilde{f},L\right)$ is an int-soft filter of *L* by Theorem 9.

**Theorem 14 .**

A soft set $\left(\tilde{f},L\right)$ over *U* is an int-soft filter of *L* if and only if the set

(41) $$\begin{array}{c} {\tilde{f}}_{\tau }∶=\left\{x\in L\;|\;\tau \subseteq \tilde{f}\left(x\right)\right\} \end{array}$$

is a filter of *L* for all *τ* ∈ *P*(*U*) with ${\tilde{f}}_{\tau }\neq \emptyset$.

**Proof**

Assume that $\left(\tilde{f},L\right)$ is an int-soft filter of *L*. Let *x*, *y* ∈ *L* and *τ* ∈ *P*(*U*) be such that $x\in {\tilde{f}}_{\tau }$ and $x\to y\in {\tilde{f}}_{\tau }$. Then $\tau \subseteq \tilde{f}(x)$ and $\tau \subseteq \tilde{f}(x\to y)$. It follows from (24) and (25) that $\tilde{f}(1)⊇\tilde{f}(x)⊇\tau$ and $\tilde{f}(y)⊇\tilde{f}(x)\cap \tilde{f}(x\to y)⊇\tau$ and so that $1\in {\tilde{f}}_{\tau }$ and $y\in {\tilde{f}}_{\tau }$. Hence ${\tilde{f}}_{\tau }$ is a filter of *L* by Proposition 4.

Conversely, suppose that ${\tilde{f}}_{\tau }$ is a filter of *L* for all *τ* ∈ *P*(*U*) with ${\tilde{f}}_{\tau }\neq \emptyset$. For any *x* ∈ *L*, let $\tilde{f}(x)=\delta$. Then $x\in {\tilde{f}}_{\delta }$ and ${\tilde{f}}_{\delta }$ is a filter of *L*. Hence $1\in {\tilde{f}}_{\delta }$ and so $\tilde{f}(x)=\delta \subseteq \tilde{f}(1)$. For any *x*, *y* ∈ *L*, let $\tilde{f}(x)=\delta_{x}$ and $\tilde{f}(x\to y)=\delta_{x\to y}$. If we take *δ* = *δ*_*x*_∩*δ*_*x*→*y*_, then $x\in {\tilde{f}}_{\delta }$ and $x\to y\in {\tilde{f}}_{\delta }$ which imply that $y\in {\tilde{f}}_{\delta }$. Thus

(42) $$\begin{array}{c} \tilde{f}\left(x\right)\cap \tilde{f}\left(x⟶y\right)=\delta_{x}\cap \delta_{x\to y}=\delta \subseteq \tilde{f}\left(y\right). \end{array}$$

Therefore $\left(\tilde{f},L\right)$ is an int-soft filter of *L* by Theorem 9.

**Theorem 15 .**

For a soft set $\left(\tilde{f},L\right)$ over *U*, let $\left({\tilde{f}}^{*},L\right)$ be a soft set over *U*, where

(43) $$\begin{array}{c} {\tilde{f}}^{*}:L⟶P\left(U\right),\;\;x⟼\{\begin{array}{cc} \tilde{f}\left(x\right) & if\;\;x\in {\tilde{f}}_{\tau },\\ \emptyset & otherwise, \end{array} \end{array}$$

where *τ* ∈ *P*(*U*)∖{*∅*}. If $\left(\tilde{f},L\right)$ is an int-soft filter of *L*, then so is $\left({\tilde{f}}^{*},L\right)$.

**Proof**

Suppose that $\left(\tilde{f},L\right)$ is an int-soft filter of *L*. Then ${\tilde{f}}_{\tau }$ is a filter of *L* for all *τ* ∈ *P*(*U*) with ${\tilde{f}}_{\tau }\neq \emptyset$ by Theorem 14. Thus $1\in {\tilde{f}}_{\tau }$, and so ${\tilde{f}}^{*}(1)=\tilde{f}(1)⊇\tilde{f}(x)⊇{\tilde{f}}^{*}(x)$ for all *x* ∈ *L*. Let *x*, *y* ∈ *L*. If $x\in {\tilde{f}}_{\tau }$ and $x\to y\in {\tilde{f}}_{\tau }$, then $y\in {\tilde{f}}_{\tau }$. Hence

(44) f~∗(x)∩f~∗(x⟶y)=f~(x)∩f~(x⟶y)⊆f~(y)=f~∗(y).

If $x\notin {\tilde{f}}_{\tau }$ or $x\to y\notin {\tilde{f}}_{\tau }$, then ${\tilde{f}}^{*}(x)=\emptyset$ or ${\tilde{f}}^{*}(x\to y)=\emptyset$. Thus

(45) $$\begin{array}{c} {\tilde{f}}^{*}\left(x\right)\cap {\tilde{f}}^{*}\left(x⟶y\right)=\emptyset \subseteq {\tilde{f}}^{*}\left(y\right). \end{array}$$

Therefore $\left({\tilde{f}}^{*},L\right)$ is an int-soft filter of *L*.

**Theorem 16 .**

If $\left(\tilde{f},L\right)$ is an int-soft filter of *L*, then the set

(46) $$\begin{array}{c} L_{a}∶=\left\{x\in L\;|\;\tilde{f}\left(a\right)\subseteq \tilde{f}\left(x\right)\right\} \end{array}$$

is a filter of *L* for every *a* ∈ *L*.

**Proof**

Since $\tilde{f}(1)⊇\tilde{f}(a)$ for all *a* ∈ *L*, we have 1 ∈ *L*_*a*_. Let *x*, *y* ∈ *L* be such that *x* ∈ *L*_*a*_ and *x* → *y* ∈ *L*_*a*_. Then $\tilde{f}(x)⊇\tilde{f}(a)$ and $\tilde{f}(x\to y)⊇\tilde{f}(a)$. Since $\left(\tilde{f},L\right)$ is an int-soft filter of *L*, it follows from (25) that

(47) $$\begin{array}{c} \tilde{f}\left(a\right)\subseteq \tilde{f}\left(x\right)\cap \tilde{f}\left(x⟶y\right)\subseteq \tilde{f}\left(y\right) \end{array}$$

so that *y* ∈ *L*_*a*_. Hence *L*_*a*_ is a filter of *L* by Proposition 4.

**Theorem 17 .**

Let *a* ∈ *L* and let $\left(\tilde{f},L\right)$ be a soft set over *U*. Then

(1) if *L*_*a*_ is a filter of *L*, then $\left(\tilde{f},L\right)$ satisfies the following condition:
  (48) (∀x,y∈L) (f~(a)⊆f~(x)∩f~(x⟶y)⟹f~(a) ⊆f~(y));
(2) if $\left(\tilde{f},L\right)$ satisfies (24) and (48), then *L*_*a*_ is a filter of *L*.

**Proof**

(1) Assume that *L*_*a*_ is a filter of *L*. Let *x*, *y* ∈ *L* be such that

(49) $$\begin{array}{c} \tilde{f}\left(a\right)\subseteq \tilde{f}\left(x\right)\cap \tilde{f}\left(x⟶y\right). \end{array}$$

Then *x* → *y* ∈ *L*_*a*_ and *x* ∈ *L*_*a*_. Using (20), we have *y* ∈ *L*_*a*_ and so $\tilde{f}(a)\subseteq \tilde{f}(y)$.

(2) Suppose that $\left(\tilde{f},L\right)$ satisfies (24) and (48). From (24) it follows that 1 ∈ *L*_*a*_. Let *x*, *y* ∈ *L* be such that *x* ∈ *L*_*a*_ and *x* → *y* ∈ *L*_*a*_. Then $\tilde{f}(a)\subseteq \tilde{f}(x)$ and $\tilde{f}(a)\subseteq \tilde{f}(x\to y)$, which imply that $\tilde{f}(a)\subseteq \tilde{f}(x)\cap \tilde{f}(x\to y)$. Thus $\tilde{f}(a)\subseteq \tilde{f}(y)$ by (48), and so *y* ∈ *L*_*a*_. Therefore *L*_*a*_ is a filter of *L* by Proposition 4.

## 4. Int-Soft *G*-Filters

**Definition 18 (see [28]).**

A nonempty subset *F* of *L* is called a *G*-filter of *L* if it is a filter of *L* that satisfies the following condition:

(50) $$\begin{array}{c} \left(\forall x,y\in L\right)\;\left(\left(x⊙x\right)⟶y\in F⟹x⟶y\in F\right). \end{array}$$

**Definition 19 .**

A soft set $\left(\tilde{f},L\right)$ over *U* is called an int-soft *G*-filter of *L* if it is an int-soft filter of *L* that satisfies

(51) $$\begin{array}{c} \left(\forall x,y\in L\right)\;\left(\tilde{f}\left(\left(x⊙x\right)⟶y\right)\subseteq \tilde{f}\left(x⟶y\right)\right). \end{array}$$

Note that condition (51) is equivalent to the following condition:

(52) $$\begin{array}{c} \left(\forall x,y\in L\right)\;\left(\tilde{f}\left(x⟶\left(x⟶y\right)\right)\subseteq \tilde{f}\left(x⟶y\right)\right). \end{array}$$

**Lemma 20 .**

Every int-soft filter $\left(\tilde{f},L\right)$ of *L* satisfies the following condition:

(53) (∀x,y,z∈L) (f~(x⟶(y⟶z))∩f~(x⟶y) ⊆f~(x⟶(x⟶z))).

**Proof**

Let *x*, *y*, *z* ∈ *L*. Using (6) and (8), we have

(54) x⟶(y⟶z)=y⟶(x⟶z)≤(x⟶y)⟶(x⟶(x⟶z)).

It follows from Theorem 10 that

(55) $$\begin{array}{c} \tilde{f}\left(x⟶\left(y⟶z\right)\right)\cap \tilde{f}\left(x⟶y\right)\subseteq \tilde{f}\left(x⟶\left(x⟶z\right)\right). \end{array}$$

This completes the proof.

**Theorem 21 .**

Let $\left(\tilde{f},L\right)$ be a soft set over *U*. Then $\left(\tilde{f},L\right)$ is an int-soft *G*-filter of *L* if and only if it is an int-soft filter of *L* that satisfies the following condition:

(56) (∀x,y,z∈L) (f~(x⟶(y⟶z))∩f~(x⟶y) ⊆f~(x⟶z)).

**Proof**

Assume that $\left(\tilde{f},L\right)$ is an int-soft *G*-filter of *L*. Then $\left(\tilde{f},L\right)$ is an int-soft filter of *L*. Note that *x* ≤ 1 = (*x* → *y*)→(*x* → *y*), and thus *x* → *y* ≤ *x* → (*x* → *y*) for all *x*, *y* ∈ *L*. It follows from (22) that $\tilde{f}(x\to y)\subseteq \tilde{f}(x\to (x\to y))$. Combining this and (52), we have

(57) $$\begin{array}{c} \tilde{f}\left(x⟶y\right)=\tilde{f}\left(x⟶\left(x⟶y\right)\right) \end{array}$$

for all *x*, *y* ∈ *L*. Using (53) and (57), we have

(58) $$\begin{array}{c} \tilde{f}\left(x⟶\left(y⟶z\right)\right)\cap \tilde{f}\left(x⟶y\right)\subseteq \tilde{f}\left(x⟶z\right) \end{array}$$

for all *x*, *y*, *z* ∈ *L*.

Conversely, let $\left(\tilde{f},L\right)$ be an int-soft filter of *L* that satisfies condition (56). If we put *y* = *x* and *z* = *y* in (56) and use (3) and (24), then

(59) f~(x⟶y)⊇f~(x⟶(x⟶y))∩f~(x⟶x)=f~(x⟶(x⟶y))∩f~(1)=f~(x⟶(x⟶y))

for all *x*, *y* ∈ *L*. Therefore $\left(\tilde{f},L\right)$ is an int-soft *G*-filter of *L*.

**Theorem 22 .**

Let $\left(\tilde{f},L\right)$ be a soft set over *U* that satisfies condition (24) and

(60) (∀x,y,z∈L) (f~(x)∩f~((y⟶z)⟶(x⟶y)) ⊆f~(y)).

Then $\left(\tilde{f},L\right)$ is an int-soft *G*-filter of *L*.

**Proof**

If we take *z*∶ = 1 in (60) and use (3), then

(61) f~(x)∩f~(x⟶y)=f~(x)∩f~(1⟶(x⟶y))=f~(x)∩f~((y⟶1)⟶(x⟶y))⊆f~(y).

Hence $\left(\tilde{f},L\right)$ is an int-soft filter of *L* by Theorem 9. Let *x*, *y*, *z* ∈ *L*. Since

(62) $$\begin{array}{c} x⟶\left(y⟶z\right)\leq \left(x⟶y\right)⟶\left(x⟶\left(x⟶z\right)\right) \end{array}$$

by (6) and (8), we have $\tilde{f}(x\to (y\to z))\subseteq \tilde{f}((x\to y)\to (x\to (x\to z)))$ by (22). It follows from (22), (24), (25), (8), and (60) that

(63) f~(x⟶y)∩f~(x⟶(y⟶z)) ⊆f~(x⟶y)∩f~((x⟶y)⟶(x⟶(x⟶z))) ⊆f~(x⟶(x⟶z)) ⊆f~(((x⟶z)⟶z)⟶(x⟶z)) =f~(((x⟶z)⟶z)⟶(1⟶(x⟶z))) ⊆f~(x⟶z).

Therefore $\left(\tilde{f},L\right)$ is an int-soft *G*-filter of *L* by Theorem 21.

The following example shows that any int-soft *G*-filter may not satisfy condition (60).

**Example 23 .**

Let *L* : = [0,1] (unit interval). For any *a*, *b* ∈ *L*, define

(64) $$\begin{array}{c} a\vee b=\max\left\{a,b\right\},\;\;a\wedge b=\min\left\{a,b\right\},\\ a⟶b=\{\begin{array}{cc} 1 & \text{if}\;\;a\leq b,\\ b & \text{otherwise}, \end{array}\;\;a⊙b=\min\left\{a,b\right\}. \end{array}$$

Then (*L*, ∨, ∧, ⊙, →, 0,1) is a residuated lattice. Let $\left(\tilde{f},L\right)$ be a soft set over *U* defined by

(65) $$\begin{array}{c} \tilde{f}:L⟶P\left(U\right),\;\;x⟼\{\begin{array}{cc} \tau & \text{if}\;\;x\in \left[\frac{1}{2},1\right],\\ \emptyset & \text{otherwise}, \end{array} \end{array}$$

where *τ* ∈ *P*(*U*)∖{*∅*}. Then $\left(\tilde{f},L\right)$ is an int-soft *G*-filter of *L*. But it does not satisfy condition (60). For example,

(66) f~(23)∩f~((13⟶14)⟶(23⟶13)) =f~(23)∩f~(1)=τ⊈∅=f~(13).

**Proposition 24 .**

For an int-soft filter $\left(\tilde{f},L\right)$ of *L*, condition (60) is equivalent to the following condition:

(67) $$\begin{array}{c} \left(\forall x,y\in L\right)\;\left(\tilde{f}\left(\left(x⟶y\right)⟶x\right)\subseteq \tilde{f}\left(x\right)\right). \end{array}$$

**Proof**

Assume that condition (60) is valid. It follows from (24) and (3) that

(68) f~((x⟶y)⟶x)=f~(1)∩f~((x⟶y)⟶x)=f~(1)∩f~((x⟶y)⟶(1⟶x))⊆f~(x)

for all *x*, *y* ∈ *L*.

Conversely, suppose that condition (67) is valid. It follows from (6) and (25) that

(69) f~(x)∩f~((y⟶z)⟶(x⟶y)) =f~(x)∩f~(x⟶((y⟶z)⟶y)) ⊆f~((y⟶z)⟶y)⊆f~(y)

for all *x*, *y* ∈ *L*.

Combining Theorem 22 and Proposition 24, we have the following result.

**Theorem 25 .**

Every int-soft filter satisfying condition (67) is an int-soft *G*-filter.

**Proposition 26 .**

Every int-soft filter $\left(\tilde{f},L\right)$ of *L* with condition (60) satisfies the following condition:

(70) (∀x,y∈L) (f~((x⟶y)⟶y) ⊆f~((y⟶x)⟶x)).

**Proof**

Let $\left(\tilde{f},L\right)$ be an int-soft filter of *L* that satisfies condition (60) and let *x*, *y* ∈ *L*. Since *x* → ((*y* → *x*) → *x*) = (*y* → *x*)→(*x* → *x*) = (*y* → *x*) → 1 = 1, that is, *x* ≤ (*y* → *x*) → *x*, we have ((*y* → *x*) → *x*) → *y* ≤ *x* → *y* by (7). It follows from (8), (6), and (7) that

(71) (x⟶y)⟶y ≤(y⟶x)⟶((x⟶y)⟶x) =(x⟶y)⟶((y⟶x)⟶x) ≤(((y⟶x)⟶x)⟶y)⟶((y⟶x)⟶x).

Using (22), (24), (3), (6), and (60), we have

(72) f~((x⟶y)⟶y) ⊆f~((((y⟶x)⟶x)⟶y)⟶((y⟶x)⟶x)) =f~(1)∩f~(1⟶((((y⟶x)⟶x)⟶y)  ⟶((y⟶x)⟶x))) =f~(1)∩f~((((y⟶x)⟶x)⟶y)  ⟶(1⟶((y⟶x)⟶x))) ⊆f~((y⟶x)⟶x).

Hence condition (70) is valid.

**Proposition 27 .**

Every int-soft *G*-filter $\left(\tilde{f},L\right)$ of *L* with condition (70) satisfies condition (60).

**Proof**

Let $\left(\tilde{f},L\right)$ be an int-soft *G*-filter of *L* that satisfies condition (70). For any *x*, *y*, *z* ∈ *L*, we have

(73) f~(z)∩f~((x⟶y)⟶(z⟶x)) =f~(z)∩f~(z⟶((x⟶y)⟶x)) ⊆f~((x⟶y)⟶x) ⊆f~((x⟶y)⟶((x⟶y)⟶y)) ⊆f~((x⟶y)⟶y) ⊆f~((y⟶x)⟶x)

by (6), (25), (22), (8), (52), and (70). Since (*x* → *y*) → *x* ≤ *y* → *x* ≤ *z* → (*y* → *x*), it follows from (22) that $\tilde{f}((x\to y)\to x)\subseteq \tilde{f}(z\to (y\to x))$ and so from (25) that

(74) f~(z)∩f~((x⟶y)⟶(z⟶x)) ⊆f~(z)∩f~((x⟶y)⟶x) ⊆f~(z)∩f~(z⟶(y⟶x)) ⊆f~(y⟶x).

Therefore $\tilde{f}(z)\cap \tilde{f}((x\to y)\to (z\to x))\subseteq \tilde{f}(y\to x)\cap \tilde{f}((y\to x)\to x)\subseteq \tilde{f}(x)$. Hence condition (60) is valid.

**Theorem 28 .**

Let $\left(\tilde{f},L\right)$ be an int-soft filter of *L*. Then $\left(\tilde{f},L\right)$ is an int-soft *G*-filter of *L* if and only if the following condition holds:

(75) $$\begin{array}{c} \left(\forall x\in L\right)\;\left(\tilde{f}\left(x⟶\left(x⊙x\right)\right)=\tilde{f}\left(1\right)\right). \end{array}$$

**Proof**

Suppose that $\left(\tilde{f},L\right)$ is an int-soft *G*-filter of *L*. Since *x* → (*x* → (*x*⊙*x*)) = 1 for all *x* ∈ *L*, we have $\tilde{f}(x\to (x\to (x⊙x)))=\tilde{f}(1)$. It follows from (56) and (3) that

(76) f~(x⟶(x⊙x))⊇f~(x⟶(x⟶(x⊙x))) ∩f~(x⟶x)=f~(1)

and so from (24) that $\tilde{f}(x\to (x⊙x))=\tilde{f}(1)$ for all *x* ∈ *L*.

Conversely, let $\left(\tilde{f},L\right)$ be an int-soft filter of *L* which satisfies condition (75) and let *x*, *y* ∈ *L*. Since

(77) x⟶(x⟶y)=(x⊙x)⟶y≤(x⟶(x⊙x))⟶(x⟶y)

by (6) and (8), it follows from (22) that

(78) $$\begin{array}{c} \tilde{f}\left(x⟶\left(x⟶y\right)\right)\subseteq \tilde{f}\left(\left(x⟶\left(x⊙x\right)\right)⟶\left(x⟶y\right)\right). \end{array}$$

Hence, we have

(79) f~(x⟶y) ⊇f~((x⟶(x⊙x))⟶(x⟶y))∩f~(x⟶(x⊙x)) ⊇f~(x⟶(x⟶y))∩f~(x⟶(x⊙x)) =f~(x⟶(x⟶y))∩f~(1) =f~(x⟶(x⟶y))

by using (25), (75), and (24). Hence $\left(\tilde{f},L\right)$ is an int-soft *G*-filter of *L*.

**Theorem 29 .**

For an int-soft filter $\left(\tilde{f},L\right)$ of *L*, the following assertions are equivalent:

1. $\left(\tilde{f},L\right)$ is an int-soft *G*-filter of *L*;
2. $(\forall x,y\in L)\;\;\left(\tilde{f}(x\to y)=\tilde{f}(x\to (x\to y))\right)$.

**Proof**

(1)⇒(2). Suppose that $\left(\tilde{f},L\right)$ is an int-soft *G*-filter of *L* and let *x*, *y* ∈ *L*. Since *x* → *y* ≤ *x* → (*x* → *y*), it follows from (22) that $\tilde{f}(x\to y)\subseteq \tilde{f}(x\to (x\to y))$. Hence $\tilde{f}(x\to y)=\tilde{f}(x\to (x\to y))$ by using (52).

(2)⇒(1). Assume that (2) holds. Using Lemma 20 and (2), we have

(80) f~(x⟶(y⟶z))∩f~(x⟶y) ⊆f~(x⟶(x⟶z))=f~(x⟶z)

for all *x*, *y*, *z* ∈ *L*, and so $\left(\tilde{f},L\right)$ is an int-soft *G*-filter of *L* by Theorem 21.

**Proposition 30 .**

Every int-soft *G*-filter $\left(\tilde{f},L\right)$ of *L* satisfies the following conditions:

(81) (∀x,y,z∈L) (f~(x⟶(y⟶z)) ⊆f~((x⟶y)⟶(x⟶z))),

(82) (∀x,y,z∈L) (f~(x⟶(y⟶z)) =f~((x⟶y)⟶(x⟶z))).

**Proof**

Let $\left(\tilde{f},L\right)$ be an int-soft *G*-filter of *L*. Using (6), (56), (8), and (24), we have

(83) f~((x⟶y)⟶(x⟶z)) =f~(x⟶((x⟶y)⟶z)) ⊇f~(x⟶(y⟶z))  ∩f~(x⟶((y⟶z)⟶((x⟶y)⟶z))) =f~(x⟶(y⟶z))  ∩f~((y⟶z)⟶((x⟶y)⟶(x⟶z))) =f~(x⟶(y⟶z))∩f~(1) =f~(x⟶(y⟶z))

for all *x*, *y*, *z* ∈ *L*. Thus (81) holds. Since (*x* → *y*)→(*x* → *z*) ≤ *x* → (*y* → *z*) for all *x*, *y*, *z* ∈ *L*, it follows from (22) that $\tilde{f}((x\to y)\to (x\to z))\subseteq \tilde{f}(x\to (y\to z))$ and so that

(84) $$\begin{array}{c} \tilde{f}\left(x⟶\left(y⟶z\right)\right)=\tilde{f}\left(\left(x⟶y\right)⟶\left(x⟶z\right)\right) \end{array}$$

for all *x*, *y*, *z* ∈ *L* by using (81).

**Proposition 31 .**

Assume that *L* satisfies the divisibility; that is, *x*∧*y* = *x*⊙(*x* → *y*), for all *x*, *y* ∈ *L*. If $\left(\tilde{f},L\right)$ is an int-soft *G*-filter of *L* satisfying (82), then the following equality is true:

(85) $$\begin{array}{c} \left(\forall x,y,z\in L\right)\;\left(\tilde{f}\left(\left(x⊙y\right)⟶z\right)=\tilde{f}\left(\left(x\wedge y\right)⟶z\right)\right). \end{array}$$

**Proof**

Using the divisibility and (6), we have

(86) (x∧y)⟶z=(x⊙(x⟶y))⟶z=(x⟶y)⟶(x⟶z)

for all *x*, *y*, *z* ∈ *L*. It follows from (6) and (82) that

(87) f~((x⊙y)⟶z)=f~(x⟶(y⟶z))=f~((x⟶y)⟶(x⟶z))=f~((x∧y)⟶z)

for all *x*, *y*, *z* ∈ *L*.

**Theorem 32 .**

Let *L* satisfy the divisibility; that is, *x*∧*y* = *x*⊙(*x* → *y*), for all *x*, *y* ∈ *L*. Then every int-soft filter $\left(\tilde{f},L\right)$ of *L* satisfying condition (85) is an int-soft *G*-filter of *L*.

**Proof**

Using Lemma 20 and (6) and (85), we have

(88) f~(x⟶(y⟶z))∩f~(x⟶y) ⊆f~(x⟶(x⟶z)) =f~((x⊙x)⟶z)=f~((x∧x)⟶z)=f~(x⟶z)

for all *x*, *y*, *z* ∈ *L*. Therefore $\left(\tilde{f},L\right)$ is an int-soft *G*-filter of *L* by Theorem 21.

**Theorem 33 (extension property).**

Let $\left(\tilde{f},L\right)$ and $\left(\tilde{g},L\right)$ be int-soft filters of *L* such that $\left(\tilde{f},L\right)\subseteq \left(\tilde{g},L\right)$; that is, $\tilde{f}(x)\subseteq \tilde{g}(x)$ for all *x* ∈ *L* and $\tilde{f}(1)=\tilde{g}(1)$. If $\left(\tilde{f},L\right)$ is an int-soft *G*-filter of *L*, then so is $\left(\tilde{g},L\right)$.

**Proof**

Assume that $\left(\tilde{f},L\right)$ is an int-soft *G*-filter of *L*. Using (6) and (3), we have

(89) x⟶(x⟶((x⟶(x⟶y))⟶y)) =(x⟶(x⟶y))⟶(x⟶(x⟶y))=1

for all *x*, *y* ∈ *L*. Thus

(90) g~(x⟶((x⟶(x⟶y))⟶y)) ⊇f~(x⟶((x⟶(x⟶y))⟶y)) =f~(x⟶(x⟶((x⟶(x⟶y))⟶y))) =f~(1)=g~(1)

by hypotheses and (57), and so

(91) $$\begin{array}{c} \tilde{g}\left(x⟶\left(\left(x⟶\left(x⟶y\right)\right)⟶y\right)\right)=\tilde{g}\left(1\right) \end{array}$$

for all *x*, *y* ∈ *L* by (24). Since $\left(\tilde{g},L\right)$ is an int-soft filter of *L*, it follows from (25), (6), and (24) that

(92) g~(x⟶y) ⊇g~(x⟶(x⟶y))  ∩g~((x⟶(x⟶y))⟶(x⟶y)) =g~(x⟶(x⟶y))  ∩g~(x⟶((x⟶(x⟶y))⟶y)) =g~(x⟶(x⟶y))∩g~(1) =g~(x⟶(x⟶y))

for all *x*, *y* ∈ *L*. Therefore $\left(\tilde{g},L\right)$ is an int-soft *G*-filter of *L*.

## 5. Regular and *MV*-Int-Soft Filters

Zhu and Xu [29] introduced the notion of a regular filter in a residuated lattice.

**Definition 34 (see [29]).**

A filter *F* of *L* is said to be* regular* if it satisfies the following condition:

(93) $$\begin{array}{c} \left(\forall x\in L\right)\;\left(\neg \neg x⟶x\in F\right). \end{array}$$

**Definition 35 .**

An int-soft filter $\left(\tilde{f},L\right)$ of *L* is said to be* regular* if it satisfies

(94) $$\begin{array}{c} \left(\forall x\in L\right)\;\left(\tilde{f}\left(\neg \neg x⟶x\right)=\tilde{f}\left(1\right)\right). \end{array}$$

**Example 36 .**

Let *L* : = [0,1] (unit interval). For any *a*, *b* ∈ *L*, define

(95) $$\begin{array}{c} a\vee b=\max\left\{a,b\right\},\;\;a\wedge b=\min\left\{a,b\right\},\\ a⟶b=\{\begin{array}{cc} 1 & \text{if}\;\;a\leq b,\\ \left(1-a\right)\vee b & \text{otherwise}, \end{array}\\ a⊙b=\{\begin{array}{cc} 0 & \text{if}\;\;a+b\leq 1,\\ a\wedge b & \text{otherwise}. \end{array} \end{array}$$

Then (*L*, ∨, ∧, ⊙, →, 0,1) is a residuated lattice (see [29]). Let $\left(\tilde{f},L\right)$ be a soft set over *U* = [0,1] defined by

(96) $$\begin{array}{c} \tilde{f}:L⟶P\left(U\right).\\ x⟼\{\begin{array}{cc} \left(x,1\right] & \text{if}\;\;x\in \left[0.5,1\right],\\ \emptyset & \text{otherwise}, \end{array} \end{array}$$

Then $\left(\tilde{f},L\right)$ is a regular int-soft filter of *L*.

**Theorem 37 .**

For an int-soft filter $\left(\tilde{f},L\right)$ of *L*, the following assertions are equivalent.

1. $\left(\tilde{f},L\right)$ is regular.
2. $(\forall x,y\in L)\;\;\left(\tilde{f}(\neg x\to \neg y)\subseteq \tilde{f}(y\to x)\right)$.
3. $(\forall x,y\in L)\;\;\left(\tilde{f}(\neg x\to y)\subseteq \tilde{f}(\neg y\to x)\right)$.

**Proof**

Assume that $\left(\tilde{f},L\right)$ is a regular int-soft filter of *L* and let *x*, *y* ∈ *L*. Using (7) and (10), we have

(97) $$\begin{array}{c} \neg x⟶\neg y\leq \neg \neg y⟶\neg \neg x\leq y⟶\neg \neg x. \end{array}$$

It follows from (8) and (7) that

(98) ¬¬x⟶x≤(y⟶¬¬x)⟶(y⟶x)≤(¬x⟶¬y)⟶(y⟶x)

and so from (24), (94), and (25) that

(99) f~(¬x⟶¬y) =f~(¬x⟶¬y)∩f~(1) =f~(¬x⟶¬y)∩f~(¬¬x⟶x) ⊆f~(¬x⟶¬y)∩f~((¬x⟶¬y)⟶(y⟶x)) ⊆f~(y⟶x);

that is, the second condition holds. Since ¬*x* → *y* ≤ ¬*y* → ¬¬*x*, we have

(100) ¬¬x⟶x≤(¬y⟶¬¬x)⟶(¬y⟶x)≤(¬x⟶y)⟶(¬y⟶x)

by (8) and (7). It follows from (24), (94), and (25) that

(101) f~(¬x⟶y) =f~(¬x⟶y)∩f~(1) =f~(¬x⟶y)∩f~(¬¬x⟶x) ⊆f~(¬x⟶y)∩f~((¬x⟶y)⟶(¬y⟶x)) ⊆f~(¬y⟶x).

Hence the third condition holds. Next, suppose that the second condition is valid. Condition (10) together with the second condition induces

(102) $$\begin{array}{c} \tilde{f}\left(1\right)=\tilde{f}\left(\neg x⟶\neg \neg \neg x\right)\subseteq \tilde{f}\left(\neg \neg x⟶x\right) \end{array}$$

for all *x* ∈ *L*, and so $\tilde{f}(\neg \neg x\to x)=\tilde{f}(1)$. Hence $\left(\tilde{f},L\right)$ is regular. Finally, assume that the third condition is valid. Since ¬*x* → ¬*x* = 1 for all *x* ∈ *L*, it follows from (3) that $\tilde{f}(1)=\tilde{f}(\neg x\to \neg x)\subseteq \tilde{f}(\neg \neg x\to x)$, and so $\tilde{f}(\neg \neg x\to x)=\tilde{f}(1)$ by (24). Therefore $\left(\tilde{f},L\right)$ is regular.

**Theorem 38 .**

A soft set $\left(\tilde{f},L\right)$ over *U* is a regular int-soft filter of *L* if and only if it satisfies condition (24) and

(103) (∀x,y,z∈L) (f~(z)∩f~(z⟶(¬x⟶y)) ⊆f~(¬y⟶x)).

**Proof**

Assume that $\left(\tilde{f},L\right)$ is a regular int-soft filter of *L*. Clearly condition (24) holds. Using (25) and Theorem 37(3), we get

(104) f~(z)∩f~(z⟶(¬x⟶y)) ⊆f~(¬x⟶y)⊆f~(¬y⟶x)

for all *x*, *y*, *z* ∈ *L*.

Conversely, suppose that $\left(\tilde{f},L\right)$ satisfies two conditions (24) and (103). Let *x*, *y* ∈ *L*. Since *x* → *y* = *x* → (1 → *y*) = *x* → (¬0 → *y*) and ¬¬*y* = 1 → ¬¬*y* = 1 → (¬*y* → 0), it follows from (3), (24), and (103) that

(105) f~(x)∩f~(x⟶y) =f~(x)∩f~(x⟶(¬0⟶y)) ⊆f~(¬y⟶0) =f~(¬¬y) =f~(1)∩f~(1⟶(¬y⟶0)) ⊆f~(¬0⟶y) =f~(1⟶y)=f~(y).

Therefore $\left(\tilde{f},L\right)$ is an int-soft filter of *L* by Theorem 9. If we take *z*∶ = 1 in (103) and use (3) and (24), then

(106) f~(¬x⟶y)=f~(1⟶(¬x⟶y))=f~(1)∩f~(1⟶(¬x⟶y))⊆f~(¬y⟶x).

Hence $\left(\tilde{f},L\right)$ is regular by Theorem 37.

By a similar way to the proof of Theorem 38, we have the following characterization of a regular int-soft filter.

**Theorem 39 .**

A soft set $\left(\tilde{f},L\right)$ over *U* is a regular int-soft filter of *L* if and only if it satisfies condition (24) and

(107) (∀x,y,z∈L) (f~(z)∩f~(z⟶(¬x⟶¬y)) ⊆f~(y⟶x)).

**Lemma 40 (see [29]).**

Let *F* be a filter of *L*. Then the following assertions are equivalent:

1. *F* is regular;
2. (∀*x*, *y* ∈ *L*)  (¬*x* → *y* ∈ *F*⇒¬*y* → *x* ∈ *F*).

**Theorem 41 .**

A soft set $\left(\tilde{f},L\right)$ over *U* is a regular int-soft filter of *L* if and only if the set

(108) $$\begin{array}{c} {\tilde{f}}_{\tau }∶=\left\{x\in L\;|\;\tau \subseteq \tilde{f}\left(x\right)\right\} \end{array}$$

is a regular filter of *L* for all *τ* ∈ *P*(*U*) with ${\tilde{f}}_{\tau }\neq \emptyset$.

**Proof**

Assume that $\left(\tilde{f},L\right)$ is a regular int-soft filter of *L*. Let *τ* ∈ *P*(*U*) be such that ${\tilde{f}}_{\tau }\neq \emptyset$. Since $\left(\tilde{f},L\right)$ is an int-soft filter of *L*, the set ${\tilde{f}}_{\tau }$ is a filter of *L* by Theorem 14. Let *x*, *y* ∈ *L* be such that $\neg x\to y\in {\tilde{f}}_{\tau }$. Then $\tau \subseteq \tilde{f}(\neg x\to y)\subseteq \tilde{f}(\neg y\to x)$ by Theorem 37, and so $\neg y\to x\in {\tilde{f}}_{\tau }$. Hence $\left(\tilde{f},L\right)$ is regular by Lemma 40.

Conversely, suppose that ${\tilde{f}}_{\tau }$ is a regular filter of *L* for all *τ* ∈ *P*(*U*) with ${\tilde{f}}_{\tau }\neq \emptyset$. Then ${\tilde{f}}_{\tau }$ is a filter of *L*, and thus $\left(\tilde{f},L\right)$ is an int-soft filter of *L* by Theorem 14. For any *x*, *y* ∈ *L*, let $\tilde{f}(\neg x\to y)=\delta$. Then $\neg x\to y\in {\tilde{f}}_{\delta }$ which implies from Lemma 40 that $\neg y\to x\in {\tilde{f}}_{\delta }$. Hence $\tilde{f}(\neg x\to y)=\delta \subseteq \tilde{f}(\neg y\to x)$, and so $\left(\tilde{f},L\right)$ is regular by Theorem 37.

**Theorem 42 .**

For any regular filter *F* of *L*, there exist *τ* ∈ *P*(*U*)∖{*∅*} and a regular int-soft filter $\left(\tilde{f},L\right)$ of *L* such that $F={\tilde{f}}_{\tau }$.

**Proof**

Let $\left(\tilde{f},L\right)$ be a soft set over *U* defined by

(109) $$\begin{array}{c} \tilde{f}:L⟶P\left(U\right),\;\;x⟼\{\begin{array}{cc} \tau & \text{if}\;\;x\in F,\\ \emptyset & \text{otherwise}, \end{array} \end{array}$$

where *τ* ∈ *P*(*U*)∖{*∅*}. Since 1 ∈ *F*, we have $\tilde{f}(x)\subseteq \tau =\tilde{f}(1)$ for all *x* ∈ *L*. Let *x*, *y*, *z* ∈ *L*. If *z* ∈ *F* and *z* → (¬*x* → *y*) ∈ *F*, then ¬*y* → *x* ∈ *F* by Proposition 4 and Lemma 40. Hence $\tilde{f}(z)\cap \tilde{f}(z\to (\neg x\to y))=\tau =\tilde{f}(\neg y\to x)$. Suppose that *z* ∉ *F* or *z* → (¬*x* → *y*) ∉ *F*. Then $\tilde{f}(z)=\emptyset$ or $\tilde{f}(\neg x\to y)=\emptyset$, and so $\tilde{f}(z)\cap \tilde{f}(z\to (\neg x\to y))=\emptyset \subseteq \tilde{f}(\neg y\to x)$. Therefore, by Theorem 38, $\left(\tilde{f},L\right)$ is a regular int-soft filter of *L*. Obviously, $F={\tilde{f}}_{\tau }$.

**Definition 43 (see [29]).**

A subset *F* of *L* is called an *MV*-filter of *L* if it is a filter of *L* that satisfies

(110) (∀x,y∈L) (y⟶x∈F⟹((x⟶y)⟶y) ⟶x∈F).

**Lemma 44 (see [29]).**

A filter *F* of *L* is an *MV*-filter of *L* if and only if it satisfies the condition

(111)   (∀x,y∈L) (((x⟶y)⟶y) ⟶((y⟶x)⟶x)∈F).

**Definition 45 .**

A soft set $\left(\tilde{f},L\right)$ over *U* is called an *MV*-int-soft filter of *L* if it is an int-soft filter of *L* with the following additional condition:

(112) (∀x,y∈L) (f~(y⟶x) ⊆f~(((x⟶y)⟶y)⟶x)).

**Theorem 46 .**

A soft set $\left(\tilde{f},L\right)$ over *U* is an *MV*-int-soft filter of *L* if and only if it satisfies condition (24) and

(113) (∀x,y,z∈L) (f~(z)∩f~(z⟶(y⟶x)) ⊆f~(((x⟶y)⟶y)⟶x)).

**Proof**

Assume that $\left(\tilde{f},L\right)$ is an *MV*-int-soft filter of *L*. Using (25) and (112), we have

(114) f~(z)∩f~(z⟶(y⟶x)) ⊆f~(y⟶x)⊆f~(((x⟶y)⟶y)⟶x)

for all *x*, *y* ∈ *L*.

Conversely, let $\left(\tilde{f},L\right)$ be a soft set over *U* which satisfies two conditions (24) and (113). Taking *y*∶ = 1 in (113) and using (3) induce condition (25). Hence $\left(\tilde{f},L\right)$ is an int-soft filter of *L* by Theorem 9. If we take *z*∶ = 1 in (113) and use (3) and (24), then we know that $\left(\tilde{f},L\right)$ satisfies condition (112). Therefore $\left(\tilde{f},L\right)$ is an *MV*-int-soft filter of *L*.

**Theorem 47 .**

Let $\left(\tilde{f},L\right)$ be an int-soft filter of *L*. Then $\left(\tilde{f},L\right)$ is an *MV*-int-soft filter of *L* if and only if the following assertion is valid:

(115) (∀x,y∈L) (f~(((x⟶y)⟶y) ⟶((y⟶x)⟶x))=f~(1)).

**Proof**

Assume that $\left(\tilde{f},L\right)$ is an *MV*-int-soft filter of *L*. Then $\left(\tilde{f},L\right)$ is an int-soft filter of *L*, and so ${\tilde{f}}_{\tau }$ is a filter of *L* for all *τ* ∈ *P*(*U*) with ${\tilde{f}}_{\tau }\neq \emptyset$ by Theorem 14. In particular, ${\tilde{f}}_{\tilde{f}(1)}$ is a filter of *L*. Let *x*, *y* ∈ *L* be such that $y\to x\in {\tilde{f}}_{\tilde{f}(1)}$. Then

(116) $$\begin{array}{c} \tilde{f}\left(1\right)\subseteq \tilde{f}\left(y⟶x\right)\subseteq \tilde{f}\left(\left(\left(x⟶y\right)⟶y\right)⟶x\right), \end{array}$$

and so $((x\to y)\to y)\to x\in {\tilde{f}}_{\tilde{f}(1)}$. Therefore ${\tilde{f}}_{\tilde{f}(1)}$ is an *MV*-filter of *L*, and thus

(117) $$\begin{array}{c} \left(\left(x⟶y\right)⟶y\right)⟶\left(\left(y⟶x\right)⟶x\right)\in {\tilde{f}}_{\tilde{f}(1)} \end{array}$$

by Lemma 44. Hence $\tilde{f}(1)\subseteq \tilde{f}(((x\to y)\to y)\to ((y\to x)\to x))$, and this and (24) imply that $\tilde{f}(((x\to y)\to y)\to ((y\to x)\to x))=\tilde{f}(1)$.

Conversely, let $\left(\tilde{f},L\right)$ be an int-soft filter of *L* that satisfies condition (115). Using (24), (115), (6), and (25), we obtain

(118) f~(y⟶x) =f~(y⟶x)∩f~(1) =f~(y⟶x)  ∩f~(((x⟶y)⟶y)⟶((y⟶x)⟶x)) =f~(y⟶x)  ∩f~((y⟶x)⟶(((x⟶y)⟶y)⟶x)) ⊆f~(((x⟶y)⟶y)⟶x).

Therefore $\left(\tilde{f},L\right)$ is an *MV*-int-soft filter of *L*.

**Theorem 48 .**

Every *MV*-int-soft filter is regular.

**Proof**

Let $\left(\tilde{f},L\right)$ be an *MV*-int-soft filter of *L*. If we take *y*∶ = 0 in (112) and use (3), then

(119) f~(1)=f~(0⟶x)⊆f~(((x⟶0)⟶0)⟶x)=f~(¬¬x⟶x)

and so $\tilde{f}(\neg \neg x\to x)=\tilde{f}(1)$ by (22). Therefore $\left(\tilde{f},L\right)$ is a regular int-soft filter of *L*.

The converse of Theorem 48 is not true in general as seen in the following example.

**Example 49 .**

Let (*L*, ∨, ∧, ⊙, →, 0,1) be the residuated lattice which is given in Example 36. Let *F*∶ = (*c*, 1] for any *c* ∈ *L*. Note that if *c* ∈ [0.5,1] then *F* is a regular filter of *L*. But, if *c* ∈ (0.7,1] then *F* is not an *MV*-filter of *L* since 0.4 → 0.7 = 1 ∈ *F*, but ((0.7 → 0.4) → 0.4) → 0.7 = 0.7 ∉ *F*. Hence the soft set $\left(\tilde{f},L\right)$ over *U* which is given as follows,

(120) $$\begin{array}{c} \tilde{f}:L⟶P\left(U\right),\;\;x⟼\{\begin{array}{cc} U & \text{if}\;\;x\in F,\\ \emptyset & \text{otherwise}, \end{array} \end{array}$$

is an int-soft filter of *L* which is regular. But, since $\tilde{f}(0.4\to 0.7)=\tilde{f}(1)=U$ and $\tilde{f}(((0.7\to 0.4)\to 0.4)\to 0.7)=\tilde{f}(0.7)=\emptyset$, therefore $\left(\tilde{f},L\right)$ is not an *MV*-int-soft filter of *L*.

In a *BL*-algebra, that is, a residuated lattice *L* with the following two conditions:

(121) $$\begin{array}{c} \left(\forall x,y\in L\right)\;\left(x\wedge y=x⊙\left(x⟶y\right)\right),\\ \left(\forall x,y\in L\right)\;\left(\left(x⟶y\right)\vee \left(y⟶x\right)=1\right), \end{array}$$

the converse of Theorem 48 is true which is shown in the following theorem.

**Theorem 50 .**

In a *BL*-algebra *L*, the notion of an *MV*-int-soft filter coincides with the notion of a regular int-soft filter.

**Proof**

Based on Theorem 48, it is sufficient to show that every regular int-soft filter is an *MV*-int-soft filter. Let $\left(\tilde{f},L\right)$ be a regular int-soft filter of *L* and let *x*, *y* ∈ *L*. Then $\tilde{f}(\neg x\to \neg y)\subseteq \tilde{f}(y\to x)$ by Theorem 37. Since *y* → *x* ≤ ¬*x* → ¬*y*, we have $\tilde{f}(y\to x)\subseteq \tilde{f}(\neg x\to \neg y)$ by (22). Hence

(122) f~(y⟶x) =f~(¬x⟶¬y)=f~(¬x⟶(¬x⟶¬y)) =f~(¬x⟶(¬y⊙(¬y⟶¬x))) =f~(¬x⟶(¬y⊙(x⟶¬¬y))) =f~(¬(¬y⊙(x⟶¬¬y))⟶x) =f~(((x⟶¬¬y)⟶(¬y⟶0))⟶x) =f~(((x⟶¬¬y)⟶¬¬y)⟶x),f~(1)=f~(¬y⟶¬y)=f~(¬¬y⟶y)⊆f~((x⟶¬¬y)⟶(x⟶y))⊆f~(((x⟶y)⟶¬¬y) ⟶((x⟶¬¬y)⟶¬¬y))⊆f~((((x⟶¬¬y)⟶¬¬y)⟶x) ⟶(((x⟶y)⟶¬¬y)⟶x)).

It follows that

(123) f~(y⟶x) =f~(y⟶x)∩f~(1) ⊆f~(((x⟶¬¬y)⟶¬¬y)⟶x) ∩f~((((x⟶¬¬y)⟶¬¬y)⟶x)⟶(((x⟶y)⟶¬¬y)⟶x)) ⊆f~((((x⟶y)⟶¬¬y)⟶x)) ⊆f~(((x⟶y)⟶y)⟶x).

Therefore $\left(\tilde{f},L\right)$ is an *MV*-int-soft filter of *L*.

## 6. Conclusions

We have introduced the notions of int-soft filters, int-soft *G*-filters, regular int-soft filters, and *MV*-int-soft filters in residuated lattices and have investigated their relations and properties. We have considered characterizations of int-soft filters, int-soft *G*-filters, regular int-soft filters, and *MV*-int-soft filters. We have provided conditions for an int-soft filter to be an int-soft *G*-filter, a regular int-soft filter, or an *MV*-int-soft filter. We have established the extension property for an int-soft *G*-filter and have shown that the notion of an *MV*-int-soft filter coincides with the notion of a regular int-soft filter in *BL*-algebras. Future research will focus on applying the notions/contents to other algebraic structures.
